# Supplementary material for: lnc-IL7R Expression Reflects Physiological Pulmonary Function and Its Aberration Is a Putative Indicator of COPD
Source: Biomedicines. 2022 Mar 28;10(4):786. doi: 10.3390/biomedicines10040786 (PMC9031132; doi:10.3390/biomedicines10040786)
Supplement: Supplementary file 1 [file biomedicines-10-00786-s001.zip › biomedicines-1629093-supplementary.pdf]

Supplementary Table S1. Association of lnc-IL7R, with GOLD COPD severity, and pulmonary function in our cohort (n = 125)

Univariate			Multivariate, correlation coefficient (95% CI)								
**Variable**	**N**	**Mean (95% CI)**	**FEV1(%)**	**FEV1(L)**	**FVC(%)**	**FVC(L)**	**post-BD_FEV1/FVC**	**Total LAA(%)**	**%LAA-950insp**	**lnc-IL7R**	**COPD severity (GOLD)**
Sex	125	0.89 ± 0.32 (0.00 − 1.00)	0.19 (0.01− 0.35)	0.30 (0.13 − 0.45)	0.23 (0.05 − 0.39)	0.36 (0.20 − 0.51)	0.05 (−0.13 −0.22)	−0.05 (−0.22 − 0.13)	−0.08 (−0.25 − 0.10)	−0.003 (−0.19 − 0.19)	−0.22 (−0.43 −0.02)
Age	125	69.09 ± 7.76 (41.00− 87.00)	−0.02 ((−.19 −0.16)	−0.31 (−0.46− −0.14)	−0.05 (−0.22 − 0.13)	−0.39 (−0.53 − −0.23)	−0.06 (−0.23 −0.12)	0.04 (−0.14 − 0.21)	0.11 (−0.07 − 0.28)	−0.09 (−0.27 − 0.10)	−0.02 (−0.26 −0.22)
BMI	122	23.54 ± 4.07 (15.80 −36.20)	0.21 (0.04 − 0.38)	0.27 (0.10 − 0.43)	0.10 (−0.07 − 0.28)	0.18 (0.00 − 0.35)	0.30 (0.13 −0.45)	−0.46 (−0.59 − 0.30)	−0.47 (−0.60 − −0.32)	−0.08 (−0.11− 0.27)	−0.41 (−0.59 − −0.19)
Smoking_Hx	125	1.384 ± 0.63 (0.00− 2.00)	−0.11 (−0.28 − 0.07)	−0.15 (−0.31− 0.03)	−0.01 (−0.18 − 0.17)	−0.10 (−0.28 − 0.07)	−0.23 (−0.39 − − 0.05)	0.26 (0.09 −0.42)	0.26 (0.09 − 0.42)	−0.11 (−0.29 − 0.08)	0.11 (−0.13 −0.34)
pack-year	125	48.98 ± 35.97 (0.00− 180.00)	−0.03 (−0.20 − 0.15)	0.02 (−0.15− 0.20)	−0.01 (−0.18 − 0.17)	0.08 (−0.09 − 0.26)	−0.05 (−0.22 −0.13)	0.17 (−0.01−0.33)	0.17 (−0.01 −0.34)	−0.04 (−0.23 − 0.15)	−0.02 (−0.26 −0.22)
FEV1 (%)	125	56.26 ± 19.16 (17.50− 97.70)		0.86 (0.80 − 0.90)	0.86 (0.80 − 0.90)	0.68 (0.58 − 0.77)	0.77 (0.69 −0.83)	−0.39 (−0.53 − − 0.23)	−0.26 (−0.42 − −0.09)	0.52 (0.37 −0.65)	−0.92 (−0.95 − −0.88)
FEV1 (L)	125	1.41 ± 0.55 (0.43 − 3.07)			0.70 (0.59 − 0.78)	0.89 (0.85 − 0.92)	0.74 (0.65 −0.81)	−0.41 (−0.55 − − 0.25)	−0.29 (−0.45 − −0.13)	0.37 (0.20 −0.53)	−0.78 (−0.86 − −0.67)
FVC(%)	124	79.80 ± 18.47 (38.50− 117.70)				0.71 (0.61− 0.79)	0.37 (0.21−0.51)	−0.15 (−0.32 − 0.03)	−0.09 (−0.27 − 0.08)	0.45 (0.28 −0.59)	−0.83 (−0.90 − −0.74)
FVC(L)	124	2.51 ± 0.71 (1.20− 4.61)					0.41 (0.25 −0.55)	−0.23 (−0.39 − − 0.05)	−0.15 (−0.32 − 0.03)	0.31 (0.13 −0.47)	−0.69 (−0.80 − −0.54)
post-BD_FEV1/FVC	125	54.66 ± 10.45 (27.00 −69.72)						−0.57 (−0.68 − − 0.44)	−0.41 (−0.55 −	0.41 (0.24 −0.55)	−0.66 (−0.77 − −0.49)

									−0.25)		
Total LAA (%)	125	14.71 ± 8.64 (0.41−45.65)							0.82 (0.75 − 0.87)	−0.35 (−0.50 −−0.17)	0.38 (0.15 − 0.56)
%LAA-950insp	125	1.34 ± 0.73 (0.00−2.00)								−0.30 (−0.46 −−0.12)	0.30 (0.06 − 0.50)
lnc-IL7R	108	0.54 ± 0.26 (0.005−1.23)									−0.59 (−0.74 −−0.38)
COPD severity_GOLD	67	1.51 ± 101.0 (0.00−3.00)									

COPD, chronic obstructive pulmonary disease; GOLD, Global Initiative for Chronic Obstructive Lung Disease; M, male; F, female; FEV1, forced expiratory volume in 1 s; FVC, forced vital capacity; BMI, body mass index; Hx, history; Post-BD, post-bronchodilator; LAA, low attenuation area; $\%LAA_{-950insp}$, percentages of low attenuation area below − 950 Hounsfield units; GOLD, Global Initiative for Chronic Obstructive Lung Disease. The values of FEV_1/FVC % and FEV_1 % were analyzed by Kruskal-Wallis tests and Dunn's multiple comparisons

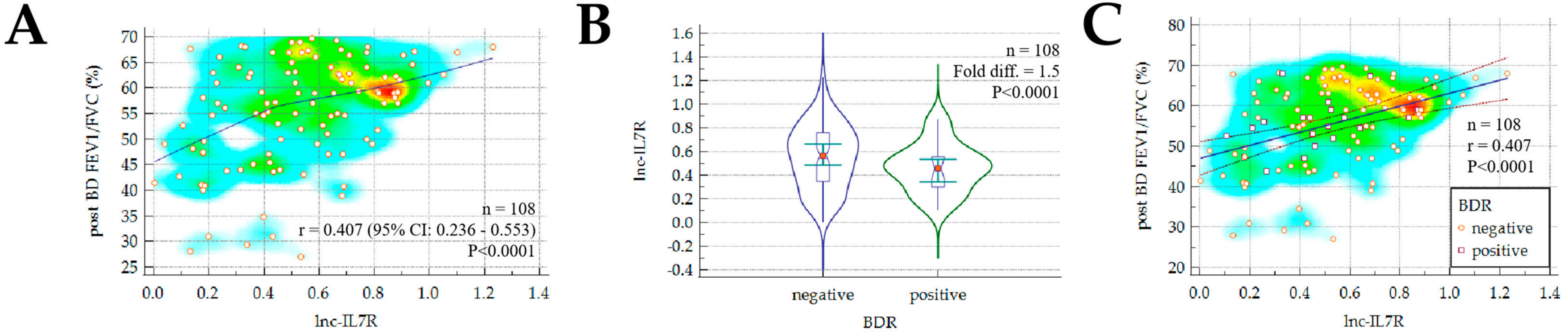

D

Regression

Dependent Y	post_BD_FEV1_FVC__%_<br>post BD FEV1/FVC (%)
Independent X	lnc_IL7R<br>lnc-IL7R

Least squares regression

All cases

Sample size	108
Coefficient of determination R^2	0.1654
Residual standard deviation	9.4755

Regression Equation

y = 47.0116 + 16.1211 x

Parameter	Coefficient	Std. Error	95% CI	t	P
Intercept	47.0116	2.0909	42.8662 to 51.1570	22.4840	<0.0001
Slope	16.1211	3.5167	9.1488 to 23.0933	4.5841	<0.0001

Analysis of Variance

Source	DF	Sum of Squares	Mean Square
Regression	1	1886.7906	1886.7906
Residual	106	9517.3152	89.7860

F-ratio	21.0143
Significance level	P<0.0001

Residuals

Chi-squared test for Normal distribution	accept Normality (P=0.8029)<br>(Chi-squared=8.594 DF=13)

Subgroup: BDR = negative

Sample size	88
Coefficient of determination R^2	0.1700
Residual standard deviation	10.1639

Regression Equation

y = 46.3389 + 16.9432 x

Parameter	Coefficient	Std. Error	95% CI	t	P
Intercept	46.3389	2.4767	41.4154 to 51.2624	18.7101	<0.0001
Slope	16.9432	4.0366	8.9188 to 24.9677	4.1974	0.0001

Analysis of Variance

Source	DF	Sum of Squares	Mean Square
Regression	1	1820.0659	1820.0659
Residual	86	8884.1782	103.3044

F-ratio	17.6185
Significance level	P=0.0001

Subgroup: BDR = positive

Sample size	20
Coefficient of determination R^2	0.1546
Residual standard deviation	5.7286

Regression Equation

y = 50.0128 + 11.7084 x

Parameter	Coefficient	Std. Error	95% CI	t	P
Intercept	50.0128	3.2435	43.1986 to 56.8270	15.4196	<0.0001
Slope	11.7084	6.4546	-1.8522 to 25.2691	1.8140	0.0864

Analysis of Variance

Source	DF	Sum of Squares	Mean Square
Regression	1	107.9815	107.9815
Residual	18	590.7006	32.8167

F-ratio	3.2904
Significance level	P=0.0864

Supplemetary Figure S1. Correlation between lnc-IL7R and bronchodilator response. (**A**) Sactter diagram showing the correlation between post-BD FEV1/FVC (%) and lnc-IL7R expression in our COPD cohort. (**B**) Combined notched box-and-whisker and violin plots comparing lnc-IL7R expression profile in patients with negative and positive BDR. (**C**) Scatter diagram with regression line showing the correlation between BDR-delineated post-BD FEV1/FVC (%) and lnc-IL7R levels in our COPD cohort. (**D**) Statistical chart showing the regression-based estimation of relationships between post-BD FEV1/FVC (%), BDR status, and lnc-IL7R ex-pression levels in our cohort.
